# Network analysis of a COVID-19 outbreak in the west of Ireland in February 2021

**DOI:** 10.1093/eurpub/ckac129.345

**Published:** 2022-10-25

**Authors:** C Timoney, B Smyth

**Affiliations:** Department of Public Health, HSE West, Health Service Executive, Galway, Ireland

## Abstract

Notification and contact tracing systems of COVID-19 hold a vast amount of information on transmission chains of the virus. It can be hard to gain an understanding of these due to the number of individuals involved. Network analysis can be used as a method of visualising these systems, gaining understanding of transmission chains and as a potential tool for monitoring outbreaks. It can link cases together and show how far reaching initial infections were. Here, a system developed in the programming language R links the Irish infectious disease notification system and contact tracing system together and creates a network representation of the result. The system finds any cases or close contacts linked in any manner to the known cases. The result is a network from the earliest found case to the latest found case or contact related to the outbreak. A large outbreak of COVID-19 occurred in the student population in the West of Ireland in February 2021 with 449 cases linked to it by the Department of Public Health at the time. Using the system, 192 further positive cases were found to be linked to the outbreak. A total of 1,431 individuals were linked in some manner to it with 68% in the 19-24 age group and less than 1% in the 65+ age group. This takes a matter of seconds to run and highlights clusters within the outbreak, the largest of which had 96 cases and 121 not detected close contacts. Visualising the transmission chains also showed that there were no other large clusters outside of the outbreak at the time. A system such as this can link cases to outbreaks not previously linked, dramatically reduce the time taken to link cases together and visualise transmission chains to gain a deeper understanding of what is happening. This automated system frees up resources to allow for deeper investigation into cases and situations of concern. It also has the potential to link outbreaks together and spot previously unnoticed situations of concern.

**Key messages:**

• Network analysis is beneficial for the monitoring of the spread of an infectious disease like COVID-19.

• Here, 192 cases were found to be linked to an outbreak that was thought to have 449 cases in it.

